# The relationship between attentional processing of emotional information and personality: A comparison between older and younger adults

**DOI:** 10.1371/journal.pone.0217382

**Published:** 2019-05-23

**Authors:** Priska Steenhaut, Ineke Demeyer, Gina Rossi, Rudi De Raedt

**Affiliations:** 1 Department of Clinical and Life Span Psychology, Vrije Universiteit Brussel (VUB), Brussels, Belgium; 2 Department of Experimental-Clinical and Health Psychology, Ghent University, Ghent, Belgium; Temple University, UNITED STATES

## Abstract

Older adults have been found to focus more on positive and less on negative information compared to younger adults. Yet, results on this attentional positivity effect are inconsistent. Since personality has been related to attentional processing in younger adults, we explored whether (mal)adaptive personality traits are also linked to the occurrence of the positivity effect measured with eye tracking paradigms. We performed two studies with different experimental tasks and recruited for each study 60 community dwelling younger (aged 24–50) and 60 older (age 65–91) adults. We found some indication for a positivity effect with a free-viewing task (study 2), but not with a task measuring engagement and disengagement with emotional information (study 1). Although this effect should be interpreted with caution, it corroborates evidence that the positivity effect is more robust in situations without cognitive constraints. No evidence was found for personality traits to be related to the occurrence of the effect. Further research is needed to further clarify conditions that influence older adults’ attention for emotional information.

## Introduction

The last decades, an extensive amount of research has focused on the occurrence of the positivity effect in healthy older adults [1]. Older adults tend to focus their attention more on positive and/or less on negative information compared to younger adults [2]. In the current literature, the most often used age for defining older adults is from 65, but mean ages in previous studies ranged from as low as 64.4 years to as high as 81.1 years, with age ranges from 60 to 93. For younger adults, most often student samples have been used, although a few studies also used slightly older, community-dwelling younger adults (age range from 18 to 39) and found positivity effects in the older adult groups in comparison with the younger group, with distraction tasks, viewing tasks and memory recall [3, 4, 5]. However, several studies failed to replicate the positivity effect [6] and effect sizes differ over studies. Recently, researchers began to investigate under which circumstances the effect does or does not occur and what the possible influencing factors may be. In our study we will focus on the positivity effect in attention and our first aim was to replicate the positivity effect in older adults using two different attentional tasks (engagement-disengagement task and a free-viewing task). However, our major aim was to explore whether personality may be a possible factor related to the occurrence of the effect, given that personality and attention are found to be related in younger adult samples, and that personality changes with ageing.

### Positivity effect

Despite an increased probability of being confronted with losses, older adults experience at least as much or even more positive and less negative affect compared to younger adults [7]. The most prominent explanation for these intriguing findings stems from socio-emotional selectivity theory [8]. It states that an age-related limited timespan leads to a shift in priorities from gaining knowledge to solve future problems towards maintaining one’s well-being at the short term. Consequently, older adults allocate more resources towards emotion regulation strategies, such as attentional deployment (shifting of attention to certain aspects of a situation in order to influence one’s emotions, and thus well-being [9]). This shift may also explain why the positivity bias occurs in older and not in younger adults, although literature is not clear on when exactly in the lifespan this motivational shift would occur. In the meta-analysis of Reed and colleagues [2] it is suggested that the positivity effect occurs gradually over the lifespan, which has been empirically confirmed for ratings of emotional information [10], but to our knowledge, this has not yet been investigated with tasks measuring attention for emotional stimuli. If the positivity effect indeed occurs gradually over the lifespan, next to differences between age cohorts, we would expect to also find correlations of the positivity or negativity bias with age within age groups with a sufficiently large age range (e.g. in a sample from +/- 25–50 years and in a sample from +/- 65–90 years). In current studies, mostly undergraduates with a limited age range have been used as young comparison groups, which is also a cohort with very specific characteristics. Therefore, in our study, we used age groups of the general population ranging from 25 to 50 (25–49), and + 65 (65–91). This allows us to not only investigate differences between age cohorts with a sufficiently large age gap (15 years of difference between groups), but also within group correlations.

### Factors influencing the occurrence of the positivity effect

In their meta-analysis, Reed and colleagues concluded that under certain conditions the effect size of the positivity effect is smaller than under other conditions (e.g. no cognitive constraints on processing and larger age differences between groups lead to a stronger effect) [2]. Therefore, in our project we ran two studies with different tasks, one with and one without constraints. Other studies focused on mood as an influencing factor. Given that the positivity effect is considered to be an emotion regulation strategy, it has been tested whether the effect would be more pronounced during an emotional state in which emotion regulation is needed. Using negative mood induction during a natural viewing task, older adults indeed show gaze preferences towards positive and away from negative faces, indicating that they gazed in a mood-repairing way [11]. They showed no gaze preference when in a positive or neutral mood. Similar results were found in older adults with an attentional engagement-disengagement task after a stress-induction: increased sad mood was related to slower attentional disengagement from happy faces [12]. However, these results contrast with the mood-congruent attentional pattern that has been found in younger adults (i.e. gaze preference towards negative and away from positive faces during negative mood [11]).

### Personality and attention

The above-mentioned mood-congruent attentional pattern in younger adults is very similar to the personality trait-congruent relations theorized to exist, that have been found in younger adult studies. There is good reason to believe that personality traits would play an important role in attentional processing, given that emotional processing is a central feature of personality characteristics [13]. The theoretically most strongly related personality traits to emotionality are known to be neuroticism and extraversion. According to Gray’s reinforcement sensitivity theory [14] people scoring higher on neuroticism are more sensitive to punishment, which could make them more attentive to negative stimuli, compared to individuals scoring lower on neuroticism. More extraverted persons, on the other hand, are more sensitive to reward, compared to individuals scoring higher on introversion. Consequently, they would be more attentive towards positive stimuli. Using attention and memory tasks, it has been found in younger adults that negative personality traits such as neuroticism, negative emotionality and avoidance temperament (i.e. a combination of neuroticism, trait negative affect and behavioural inhibition) are positively related to the processing of negative emotional stimuli (e.g. faster recognition of negative words, more maintained attention, etc [15, 16, 17]). On the other hand, more positive traits such as extraversion, positive emotionality and approach temperament have been positively associated with the processing of positive emotional stimuli [15, 16, 17]. Although effect sizes were rather small, and did not always reach significance [15, 16, 17], it was found that when investigating both personality and naturally occurring mood, personality was the only predictor of emotional information processing and thus had a stronger influence than mood [18]. So, although, evidence in younger adults shows that personality traits play a role in emotional information processing, this has not yet been investigated in older adults, and studies investigating the relationship between personality traits and the age-related positivity effect in attention are scarce. However, this may be important because it is known that personality slightly changes over the life span, with neuroticism, extraversion and openness decreasing with ageing and conscientiousness and agreeableness increasing [19]. In one study, investigating valence judgements, it was found that agreeableness and conscientiousness were related to more positive rating of emotional stimuli in an adult group with age range of 19 to 69 years [10]. However, the relationship between personality traits and attention to emotional stimuli has not yet been investigated in an older adult population. If personality traits and attentional processing are related throughout the lifespan and personality changes with ageing, it might be that the age-related change in attentional bias is moderated by personality. For example, neuroticism is related to a negativity bias in younger adults. However, neuroticism declines with ageing, so the attentional bias might reduce as well. If this were the case, it might provide a partial explanation of why older adults focus more on the positive information, compared to younger adults.

### Current study

Therefore, besides trying to corroborate the positivity effect under different attention conditions, we investigated the relationship between personality traits and attentional processing of emotional stimuli in older adults compared to younger adults.

Based on the theoretical relevance of attention for emotional information to specific personality characteristics and the results of earlier studies [15, 16, 17, 18], we mainly focused on neuroticism and extraversion. Because psychopathology and personality disorders are strongly associated with dysfunctional emotion regulation [20], we also looked at the maladaptive variants of the Personality Psychopathology Five-Revised (PSY-5-r [21]): negative emotionality and low positive emotionality. We additionally included conscientiousness in the results section, given that the positivity effect may be a result of different use the regulation strategy attentional deployment by older compared to younger adults. Consequently, it might be that a regulatory trait as conscientiousness might also play a role the occurrence of this effect. Unlike most of the younger adult studies described above, we did not use aggregated personality measures (e.g. neuroticism + trait negative affect + behavioural inhibition) for two reasons. First, in clinical practice these aggregated personality measures are not used either, so it is of more interest to investigate how the separate constructs relate to emotional information processing. Secondly, non-aggregated traits may provide more detailed information about the relation between personality and attention.

Most studies described above concerning personality and attention were limited to student samples from a younger adult population, yet these are not representative for the whole younger adult population. Therefore, in both studies we included a more natural reference group of adults between the ages of 25 and 50 years old from different educational levels (i.e. not only students). For our older adult group, we recruited persons above the age of 65, which is the most used cut-off to define older adults.

## Study 1: Engagement-disengagement task

This task allows us to further clarify the mechanisms driving the positivity effect, as it measures distinct mechanisms of attention (i.e. engagement towards and disengagement from emotional stimuli). More specifically, the positivity effect we expected to replicate in older adults might constitute faster engagement towards happy faces, and/or slower disengagement (more maintained attention) for happy faces, and/or slower engagement towards sad and/or faster disengagement (less maintained attention) for sad faces compared to younger adults. Moreover, within-groups, we expected positive correlations between age and disengagement for happy faces (the higher the age, the higher the reaction time, thus the slower looking away from happy faces) and engagement to sad faces (the older, the slower looking towards sad faces). We anticipated negative correlations between age and engagement towards happy faces (the older, the faster looking at happy faces) and disengagement from sad faces (the higher the age, the faster looking away from sad faces).

Secondly, in line with previous studies [15, 16, 17, 18], in younger adults we expected extraversion and positive emotionality to be related to faster engagement to, and/or slower disengagement from happy faces, and neuroticism and negative emotionality to be associated with faster engagement to and/or slower disengagement from sad faces. The relationship between conscientiousness and information processing has been less empirically investigated, but based on the study investigating valence judgement [10], we would expect conscientiousness to be related to faster engagement to, and/or slower disengagement from happy faces. Moreover, we explored whether the relationship between attention and personality characteristics for older adults would be different as compared to younger adults (moderation of personality in the age-attention relationship). In line with the literature on mood-incongruent attentional patterns in older adults [11, 12], we expected that, as compared to younger adults, older adults scoring higher on neuroticism and negative emotionality would apply an emotion regulation mechanism resulting in an attentional preference for positive over negative information (i.e. the positivity effect in attention).

## Method

This study was part of a larger older adult project approved by the ethical committee of the Faculty of Psychology and Educational Sciences of Ghent University. Written informed consent was obtained from all participants.

### Participants

We recruited 60 younger (25–50 years) and 60 older adults (65+) from a Dutch-speaking community sample with flyers, advertisement, social media, in recreational clubs and at education centres for seniors. Before testing, we screened over the telephone: Persons who reported uncorrected vision problems, known cognition problems (i.e. dementia) or a diagnosis of psychological illnesses during the last five years were excluded from participating. Due to technical failure of the eye-tracker, data of two older participants could not be recorded. Five younger and seven older participants who did not have at least 50% of their eye-tracking data registered (due to problems with lenses, glasses or attention) and three younger and three older participants with invalid Minnesota Multiphasic Personality Inventory-2-Restructured Form (MMPI-2-RF; see Materials) profiles were excluded. Further, there was one older participant who scored too low on the Mini-Mental State Examination (MMSE; see materials) and one who scored too low on the MMSE and did not have enough eye-tracking data registered. Finally, one older participant was excluded because of too less eye-tracking data and an invalid MMPI-2-RF profile. Analyses were performed with the remaining 52 younger adults (*M*age = 32.67, *SD* = 8.28, *range* = 25–49) and 45 older adults (*M*age = 71.40, *SD* = 5.35, *range* = 65–91). Demographics for the younger and older adult group can be found in Table 1. Based on gender and marital status, the samples in this study are representative for the general population. However, they are higher educated than younger and older adults in the general population [22]. All participants are Caucasian.

**Table 1:** Study 1: Demographics, personality traits and attentional speed per age group.

Demographics	Younger adults	Older adults	*X*^*2*^	*p*	
Marital status					
Married/living together	42.4%	64.5%			
Single/divorced/widow(er)	57.7%	35.5%	4.74	.029	
Education level					
Primary school	0%	6.7%			
Secondary school	17.3%	42.2%			
Higher education	82.7%	48.9%	12.19	.002	
Gender					
Male	48.1%	53.3%			
Female	51.9%	46.7%	.27	.606	
Personality traits	YA mean score/(SD)	OA mean score/(SD)	*t*-value *df*(95)	*p*	Cohen’s *d*
Negative emotionality	7.23 (3.72)	4.78 (3.52)	3.32	.001	.68
Low positive emotionality	7.63 (3.76)	9.38 (3.30)	2.41	.018	.49
Neuroticism	-.10 (.66)	-.35 (.61)	1.93	.056	.39
Extraversion	.23 (.72)	.31 (.60)	.56	.574	.13
Conscientiousness	.43 (.53)	.61 (.57)	-1.67	.098	.33
Attention measure	YA median RT in ms/(range)	OA median RT in ms/(range)	*U*	*p*	*r*
Disengagement happy face	253 (260)	261 (370)	1038	.340	.10
Disengagement sad face	243 (550)	252 (260)	1036	.332	.10
Engagement happy face	246 (320)	261 (230)	1053	.397	.09
Engagement sad face	245 (280)	263 (290)	897	.048	.20

### Materials

#### Mini-Mental State Examination (MMSE)

The MMSE [23] (Dutch version [24]) was administered to screen for cognitive impairment in the older adult group. Scores on the MMSE range from 0 to 30, with lower scores indicating more cognitive impairment. Given that the positivity effect may not occur in older adults with limited cognitive resources like (mild) dementia [25], older adults scoring lower than 27 were excluded, following the cut-off currently used in research [26].

#### Minnesota Multiphasic Personality Inventory-2-Restructured Form (MMPI-2-RF)

The MMPI-2-RF [27] (Dutch version [28]) measures personality and psychopathology and consists of 338 statements to which participants have to respond to with ‘agree’/’do not agree’. To see whether a MMPI-2-RF profile is valid, we used the criteria for the validity scale scores [28] (pp. 36–50; i.e. TRIN < 80, VRIN < 80, F < 110, Fp < 100, L < 80 and K < 75). The scales of interest for the current study, negative emotionality, and introversion/low positive emotionality (both revised) are part of the PSY-5-r [21]. In this sample, these scales demonstrated good internal consistencies (younger adults: resp. Cronbach’s α = .76 and .75; older adults: .79 and .70).

#### Big Five Inventory (BFI)

The BFI [29] (Dutch version [30]) consists of 44 statements that can be answered on a 5-point Likert scale ranging from ‘totally disagree’ to ‘totally agree’. Scales were corrected for acquiescence [31]. In the current sample, internal consistencies were good for neuroticism, extraversion, and conscientiousness (younger adults: resp. Cronbach’s α = .82, .87, and .74; older adults: .75, .75, and .75).

#### Engagement-disengagement task

The engagement-disengagement task [32] was used to measure participant’s attentional gaze to emotional stimuli. The stimuli used were pictures of neutral and emotional (happy and sad) faces, identical to the stimuli used in [32]. A Tobii tx-300 eye-tracker system was used to record eye-movements. Participants were seated circa 65 cm/25.59 inch from the screen and were asked to keep looking at the screen to keep contact with the eye-tracking device.

In each trial a black screen (88.5 cm/34.84 inch (width) x 50.5 cm/19.88 inch (height)) was shown for 500 ms, after which a white fixation cross appeared in the centre. Once participants fixated on this cross, both a neutral and emotional face of the same person appeared centred on the screen (39 cm/15.35 inch apart from each other). Emotional faces could equally appear on the left or right side of the screen. Participants could freely watch these faces (as if they were watching tv) during a period of 3000 ms, to encourage naturalistic processing. After this free-viewing period, three conditions could occur randomly and with equal chances of occurring. In the engagement condition, the task proceeded only when fixation on the neutral face was detected for 100 ms. Then a frame appeared around the emotional face to which the participant was instructed to respond as quickly and accurate as possible by pressing ‘1’ if the frame was a rectangle or ‘2’ if it was a circle. The frames (rectangle or circle) were randomly presented and also had equal chances of occurring on the left or right side. Participants had to respond to the frame to make sure they switched their attention. The time needed to move attention (gaze) towards or ‘engage’ to the emotional face was measured. In the disengagement condition the opposite occurred. The task proceeded when fixation on the emotional face was detected for 100 ms, then a frame appeared around the neutral face and the time it took to move attentional gaze away or ‘disengage’ from the emotional face was measured. In the last condition, no frame appeared, and a new trial was started. These trials, used in other studies to calculate other indices, were not used in our further analyses.

To identify valid disengagement and engagement trials, we used the same criteria as Demeyer and colleagues [12]: 1) fixation on the opposite stimulus before the frame appeared (i.e.; detection of a valid fixation on the given stimulus during the ‘wait for fixation’ period), 2) saccades towards the framed face at least 100 ms after the frame appeared, 3) gaze was immediately directed to the stimulus surrounded by a frame (i.e., exclusion of trials with participants’ gaze remaining at the initially fixated stimulus position or other positions on the screen for more than 1000 ms after the frame appeared), and 4) fixation of at least 100 ms to the stimulus surrounded by the frame after shifting their gaze to it. In the younger adult group 87.66% of the trials was valid (SD = 10.51), in the older adult group this was 88.66%, SD = 8.48. Next, four separate attentional indices were calculated, based on how fast persons would move their gaze towards or away from a stimulus: disengagement from happy faces, disengagement from sad faces, engagement towards happy faces, and engagement towards sad faces (each index is calculated on twelve trials). They had the following internal consistencies (Cronbach’s α) in our samples: younger adults, respectively .71, .66, .64, .71 and older adults, respectively .74, .69, .73, .33. Participants completed ten practice trials, followed by 72 experimental trials (36 neutral-happy and 36 neutral-sad pairs), which were randomly presented.

### Procedure

Participants were asked to fill in the BFI, MMPI-2-RF and a biographical questionnaire at home. In the University laboratory, the written informed consent was followed by the engagement-disengagement task. The attention task started with a calibration period where participants must fixate on nine alternating points of the screen. After this, participants also performed an emotional reactivity task with physiological measures for another study of the project. After a ten minutes break, they made an internal shift task, completed well-being, and emotion regulation questionnaires, also for another study. At the end of the experiment, the older adults also completed a MMSE interview. Finally, all participants were debriefed and were payed 20 euro’s as expense compensation.

## Results

### Personality measures

Differences in personality scores between the younger and older adult group were examined with independent *t*-tests and Cohen’s *d* effect sizes. Effect sizes were considered large when higher than .80, medium above .50 and small when higher than .20 (pp. 24–27 in [33]). Older adults scored significantly lower on negative emotionality (medium effect size) and significantly lower on positive emotionality (small effect; see Table 1).

### Replication of the positivity effect

Because the scores on the ‘disengagement from sad’ index in the younger adult group (skewness = 3.28, kurtosis = 14.75) and those on the ‘engagement towards sad’ index in the older adult group (skewness = 2.40, kurtosis = 7.40) were skewed and normalization methods were not successful in removing the skewness, we applied non-parametric tests. Whether younger and older adults differed on attentional indices and whether the positivity effect in older adults could be replicated was explored with Mann-Whitney *U* tests. For the effect sizes, we transformed into *z* scores to calculate *r* (= Z/√N). *r* is considered large when higher than .50, medium above .30 and small above .10 (pp. 78–81 in [33]). Older adults were found to engage slower towards negative information compared to younger adults (small effect size). There were no significant differences in the other attentional indices (see Table 1). For the within-group correlations between age and attentional gazing we used Spearman correlations. These were not significant in the younger (all *r* < .24, *p* >.119) and older adult group (all *r* < .23, *p* > .130).

### Relationship of attention and personality in younger and older adults

Spearman correlations were used to explore the relationships between the personality measures and attentional indices. No significant correlations were found in the younger adult group (see Table 2). In our older adult group we only found that the traits negative emotionality and conscientiousness were associated with significantly faster disengagement from sad faces (medium effect sizes).

**Table 2:** Study 1: Correlations between personality and attention in younger and older adults^a^.

	Disengagement happy face	Disengagement sad face	Engagement happy face	Engagement sad face
Younger adults				
NEGE^b^	.13 (.364)	**-.06 (.701)**	-.01 (.921)	**.11 (.459)**
LowPOSE^b^	.21 (.136)	**.01 (.934)**	.03 (.836)	**-.02 (.896)**
Neuroticism	.16 (.269)	**.12 (.417)**	.07 (.624)	**.21 (.140)**
Extraversion	**-.08 (.597)**	.05 (.745)	**.12 (.415)**	.04 (.772)
Conscientiousness	.07 (.54)	-.03 (.859)	.12 (.386)	-.07 (.621)
Older adults				
NEGE^b^	-.29 (.055)	-.32 (.031)	-.28 (.067)	-.29 (.057)
LowPOSE^b^	-.15 (.340)	-.13 (.385)	-.08 (.595)	-.17 (.277)
Neuroticism	-.25 (.093)	-.21 (.174)	-.28 (.060)	-.24 (.111)
Extraversion	-.14 (.346)	-.15 (.317)	-.17 (.257)	-.08 (.588)
Conscientiousness	-.08 (.590)	-.31 (.038)	-.12 (.45)	.14 (.366)

^a^ *r*-values in table (*p*-values). **Bold:** relevant correlations with concrete hypotheses based on previous research in the younger adult group.

^b^NEGE = negative emotionality, lowPOSE = low positive emotionality

We also applied a non-parametric method to test whether the relationship between age group and attentional bias would be moderated by personality characteristics. A Fisher *r*-to-*z* transformation was performed to investigate whether the correlations between personality and attentional patterns in the older adults differed significantly from the correlations in the younger adults (http://vassarstats.net/rdiff.html). For the effect sizes, we used the following rule of thumb: a difference between two *z*-scores larger than .10 is a small effect size, a difference larger than .30 is a medium effect size and a difference larger than .50 is a large effect size (pp. 109–139 in [33]).

The correlations between both negative emotionality and neuroticism, and disengagement from the happy face, and the correlation between neuroticism and engagement towards the sad face, differed significantly between both age groups (see Table 3). All these correlations were positive in the younger adult group and negative in the older adult group, although none of them were significant within each age group.

**Table 3:** Study 1: Fisher r-to-z-transformations.

	Disengagement happy face	Disengagement sad face	Engagement happy face	Engagement sad face
NEGE^b^	.43 (.041)	.27 (.197)	.28 (.187)	.41 (.051)
LowPOSE^b^	.36 (.084)	.14 (.503)	.11 (.603)	.15 (.472)
Neuroticism	.42 (.048)	.33 (.112)	.36 (.089)	.46 (.029)
Extraversion	.06 (.772)	.20 (.337)	.29 (.165)	.12 (.569)
Conscientiousness	.15 (.478)	.29 (.168)	.25 (.250)	.21 (.317)

*Note*. *q*-value (*p*-value)

## Discussion

### Positivity effect

We found that older adults engage their attention slower towards sad faces, compared to younger adults. This seems to confirm a positivity effect. However, this effect must be evaluated as a possible age-by-valence interaction and median reaction times for engagement towards happy faces were nearly the same as the reaction times for engagement towards sad faces in both age groups (see Table 1). Therefore, it is highly unlikely that an interaction effect would be present and that the above mentioned significant difference reflects a positivity effect. Moreover, we did not find significant within-group correlations between age and attention to emotional stimuli. A possible explanation for not finding the effect in the present study is that the engagement-disengagement task puts too high constraints on the viewing process. To correctly perform the task, participants were obliged to switch their attention towards the frames. Such constraints are known to reduce the size of the effect [2]. Additionally, the engagement towards sad faces index had a low reliability in our older adult group, so results must be interpreted with caution. Moreover, the mean age gap between our groups, 38.73 years, is rather small compared to the age differences of studies used in the meta-analysis of Reed and colleagues (see Table 1 in [2]). This also reduced our likelihood of finding the positivity effect: the smaller the age difference between younger and older samples, the smaller the positivity effect [2].

### Personality and attention

Unexpectedly, we did not find evidence for a trait-congruent attentional pattern in our younger adult group. Previous studies frequently mentioned that the effect sizes for the relationship between personality and attention were rather small. Often one-tailed *p*-values were used and several times results did not reach significance, unless data were combined over several studies [15]. Taken that into consideration, it is not surprising that we did not find significant results given our rather small sample size. However, when looking at the directions of our relevant correlations with an effect size above .10 (see Table 2), we can see that four out of five are not even in the expected directions, whereas in previous studies non-significant relevant results were almost always in the expected directions [15, 16, 17, 18].

In our older adult group, we expected non-congruent patterns to occur for the negative personality traits, in line with the earlier mood studies [11, 12]. Two significant results were found (in line with expectations): higher negative emotionality and higher conscientiousness were related to faster moving attention away from the sad face towards the neutral face. However, examining all correlations with neuroticism, negative emotionality and low positive emotionality above .10 (Table 2), we can see that five out of eleven correlations were in the expected directions, which is what we could expect at chance rate. Moreover, all directions, but one, in the older adults group were negative, regardless of the different traits.

Taken together, we did find some expected results in the older adult group, but given that our insignificant results seem to have quite random directions (contrary to previous studies), we cannot conclude from these results that clear relationships between personality traits and visual attention to emotional faces were present in younger or older adults.

## Study 2: Free viewing

Because constraining the viewing process (as in our first study) might reduce the size of the positivity effect [2] we conducted a new study using a naturalistic viewing task. Additionally, we applied a more objective measure to screen for pathologies (see Materials) instead of simply asking participants whether they had psychological antecedents.

We expected older adults to look longer at happy faces (relative to neutral faces) and/or less at sad faces (relative to neutral faces), compared to younger adults. Within-groups, we expected higher age to be associated to longer looking times at happy faces and to less viewing time to sad faces. Further, we hypothesized extraversion, positive emotionality, and conscientiousness to be related to longer looking times at happy faces and neuroticism and negative emotionality to be associated with longer looking times to sad faces. We also expected age-differences in these relationships between personality and attentional processing, with older adults scoring higher on the more negative traits showing an attentional preference for positive information (i.e. the positivity effect in attention as an emotion regulation strategy).

## Method

This study was part of a larger older adult project approved by the ethical committee of the Faculty of Psychology and Educational Sciences of Ghent University and the Vrije Universiteit Brussel (VUB). All participants completed an informed consent form.

### Participants

Participants were recruited in similar ways as in study 1 (*N* = 60 in each group). Those who reported uncorrected vision problems, known cognitive problems such as dementia, or current psychiatric disorders assessed with the Mini International Neuropsychiatric Interview (MINI: see materials), during telephone screening, were excluded. One older participant who scored under the cut-off on the MMSE, and two older participants who had invalid MMPI-2-RF were excluded from the analyses. An additional seven younger and five older participants were excluded given that less than 50 percent of their eye movements were registered. Consequently, analyses included 53 younger adults (*M*age = 31, *SD* = 7.84, *range* = 24–50) and 52 older adults (*M*age = 71.15, *SD* = 4.80, *range* = 65–84). Demographic variables are provided in Table 4. The samples in this study are representative for the general population based on gender and marital status but are higher educated than younger and older adults in the general population [22]. Except for one participant, who has Belgian-Turkish roots, all participants are Caucasian.

**Table 4:** Study 2. Demographics, personality traits and attentional speed per age group.

	Younger adults	Older adults	*X*^2^	*p*	
Undivided condition					
Marital status					
Married/living together	43%	65%			
Single/divorced/widow(er)	57%	35%	5.11	.024	
Education level					
Primary school	2%	6%			
Secondary school	15%	19%			
Higher education	83%	75%	1.51	.469	
Gender					
Male	51%	50%			
Female	49%	50%	.01	.923	
Personality traits	YA mean score/(SD)	OA mean score/(SD)	*t*-value (df)	*p*	Cohen’s *d*
Negative emotionality	6.47 (4.11)	5.54 (3.71)	1.22 (103)	.225	.24
Low positive emotionality	8.70 (3.91)	8.69 (3.06)	.01 (98.20)	.993	.003
Neuroticism	-.17 (.63)	-.47 (.58)	2.54 (103)	.013	.50
Extraversion	.20 (.68)	.41 (.65)	-1.65 (103)	.102	.32
Conscientiousness	.47 (.56)	.55 (.48)	-.78 (103)	.439	.15
Attention measure	YA mean relative viewing time in ms/(SD)	OA mean relative viewing time in ms/(SD)	*t*-value (df)	*p*	Cohen’s *d*
Happy index	58 (09)	59 (11)	-.81 (95.60)	.419	.10
Sad index	48 (08)	44 (12)	2.18 (103)	.032	.39

### Materials

#### Mini International Neuropsychiatric Interview 5.0.0 (MINI)

Candidate participants were screened for current psychiatric disorders, by using the MINI [34] (Dutch version [35]). This short screening interview, based on the Diagnostic and Statistical Manual Fourth Edition (DSM-IV [36]) and the International Classification of Diseases (ICD-10 [37]), has 20 yes/no questions. A current psychiatric diagnosis is excluded when a question is answered with ‘no’. When the question is answered by ‘yes’, further questions are posed to assess whether a person should be excluded.

#### BFI

See also study 1. The scales of interest for this study, neuroticism, extraversion, and conscientiousness showed good internal consistencies (resp. among younger adults, Cronbach’s α = .84, .84, and .82; among older adults: .78, .85, and .73).

#### MMPI-2-RF

See also study 1. Both scales–negative emotionality and introversion/low positive emotionality (both revised)–showed good to acceptable internal consistencies (younger adults: Cronbach’s α = .81, and .78, respectively; among older adults: .78, and .62).

#### Naturalistic viewing task

The stimuli in the task existed of pictures of happy, sad, and neutral faces and were identical to the stimuli used by Sanchez and colleagues [32]. To record eye-movements, a Tobii tx-300 eye-tracker system was used. Participants were seated circa 65 cm/25.59 inch from the screen and were encouraged not to look away from the screen during the experiment in order to not loose contact with the eye-tracking device.

Each trial started by presenting a black screen (88.5 cm/34.84 inch (width) x 50.5 cm/19.88 inch (height)) with a white fixation cross in the middle. Participants were instructed to focus on the cross and the task only advanced when they looked at the cross for at least 100 ms. Hereafter, a black screen with two faces of the same person was presented during 6000 ms. One of the faces always had an emotional expression, while the other one was neutral. They were shown in the middle of the screen, 39 cm/15.35 inch apart from each other. Emotional faces could equally appear on the left or right side of the screen and face pairs were randomly presented. Participants were told to freely look at the screen (as if they were watching television) during this period to encourage naturalistic processing. Then, a new fixation cross appeared and a new trial was started.

For the analyses, two indexes were calculated. The happy face index is the total time spent looking at the happy faces, divided by total time spent looking at both happy and neutral faces. The sad face index is the total time spent looking at the sad faces divided, divided by total time looking at both sad and neutral faces. In the present sample, the indices had good internal consistencies in the current sample (Cronbach’s α among younger adults: respectively .82 and .82, among older adults: .89 and .90). Participants completed first three practice trials, followed by 36 experimental trials (18 neutral-happy and 18 neutral-sad pairs).

### Procedure

Participants first completed a written informed consent and thereafter the personality and biographical questionnaires at home. Upon arrival at the laboratory of Ghent University, they performed the naturalistic viewing task with eye-tracker. This task started with a calibration period where participants had to fixate on nine alternating points on the screen. After the naturalistic viewing task, participants completed tasks and questionnaires related to another study: emotional Stroop task, choice task, optional ten minutes break, followed by flexibility and well-being questionnaires. After the whole experiment was completed, the older adults also completed an MMSE interview. Finally, all participants were debriefed and were payed 20 euro’s as expense compensation.

## Results

### Personality measures

Age-related differences in personality traits were assessed with independent *t*-tests and Cohen’s *d* effect sizes. Differences were found for neuroticism, with older adults scoring significantly lower than younger adults (medium effect size; see Table 4). No significant differences were found for the other personality traits.

### Replication of the positivity effect

To investigate whether the positivity effect could be replicated with the naturalistic viewing task, a 2 x 2 mixed ANOVA was used, with emotion as a within-subject variable, and age group as between-subject variable.

A significant main effect of emotion emerged, *F*(1, 103) = 51.96, *p* < .001, *η*^*2*^_*p*_ = .34. Follow-up paired samples *t*-tests in the total group showed that participants looked significantly longer at happy faces (*M* = .59, *SD* = .10) than at sad faces (*M* = .46, *SD* = .10), *t*(104) = 7.12, *p* < .001, *d*_*av*_ = 1.3.

A trend significant interaction effect was found, *F*(1, 103) = 3.01, *p* = .086, *η*^*2*^_*p*_ = .03. Since the interaction effect was trend significant, we decided to perform follow-up independent samples *t*-tests to test our hypotheses for each attentional measure between age groups (see lower part of Table 4). These analyses show that older adults looked significantly less at sad stimuli compared to younger adults (*d* = .39), whereas there was no significant difference in time spent on happy faces.

To investigate the likeliness of the positivity effect occurring gradually over ageing, we used within-group Pearson correlations. However, no significant relationships were observed, both in the younger (*r* < -.08, *p* > .596) and the older (*r* < -.08, *p* > .536) adult group.

### Relationship of attention and personality in younger and older adults

To investigate whether personality moderates the relationship between age group and attention to emotional stimuli, 2 x 2 mixed ANCOVAs were used, with emotion as a within-subject variable, age group as between subject variable, and personality as a continuous independent variable (covariate, modelled with all main and interaction effects). For each of the four traits, a separate ANCOVA was performed. A trend significant three-way interaction was found with conscientiousness, F(1, 101) = 3.84, p = .053, *η*^*2*^*p* = .037. None of the two-way interactions (personality x emotion) were significant, all *F*(1, 101) < 2.47, *p* > .119. The other three-way interactions (personality x emotion x age group) were also not significant, all *F*(1, 101) < 1.93, *p* > .167.

Given that we found a trend significant three-way interaction effect with conscientiousness, we calculated the correlations between this personality trait and the attentional indices in both age groups. In our younger adult group we found no significant correlations (all *r* < .08, *p* > .621). In our older adult group we found a negative correlation between conscientiousness and time spent looking at sad faces (*r* = -.30, *p* = .030). No significant correlation was found with the viewing at happy faces index (*r* = .14, *p* = .314).

## Discussion

### Positivity effect

With the naturalistic viewing task, we did find some evidence for a positivity effect. Older adults spent less time looking at sad faces, relative to neutral ones, compared to younger adults. Follow-up analyses to test our specific hypotheses on the positivity effect showed a significant effect, however, the interaction was only trend-significant. Given that this effect should therefore be interpreted with caution, we cannot conclude that the existence of a positivity effect was corroborated. Moreover, contrary to the meta-analysis of Reed and colleagues [2], we did not observe evidence for a negativity bias in younger adults. The viewing duration at sad faces were in both groups significantly lower than the duration of attending the happy stimuli (contrary to findings in study 1). The positivity effect was also not driven by increased attention to positive information on the older adults’ part, but mainly from less attention to negative information. This is consistent with some previous studies e.g. [38], although evidence for increased attention to positive information also exists [2]. Based on our within-group correlations, we did not find evidence for a positivity bias in attention occurring gradually over age.

### Personality and attention

We found that conscientiousness was related to less looking at sad faces in our older adult group, but not in our younger adult group. Effects on the attentional indices with the other personality traits were found in neither age group. In the valence judgements study mentioned in the introduction [10] conscientiousness was also related to more positive processing of emotional stimuli in an age group ranging from 19 to 69 years old. It thus seems that this regulatory trait has an influence on the more positive/less negative processing of information, and that this influence is stronger in older adults compared to younger adults, although this has to be confirmed in further research. Remarkable is also that no results were found with the more affective personality traits, although these are mainly investigated when it comes to emotional information processing.

## General discussion

We aimed at corroborating a positivity effect in older adults compared to younger adults and exploring whether personality is related to attentional gaze patterns for emotional stimuli in older and younger adults in similar or different ways. To investigate these aims, two different attentional tasks were applied; an engagement-disengagement task (which constrained attention) and a free-viewing task.

### Positivity effect

We did not succeed in convincingly replicating the occurrence of the positivity effect. However, there was a trend significant interaction effect with the free-viewing task, but it is unlikely that the positivity effect we observed with the engagement-disengagement task reflects an age-by-valence interaction. Therefore, we were not able to determine in more detail the underlying mechanisms driving the effect; for example, whether it stems from trying to avoid the negative information from the beginning (less engagement towards sad stimuli) or from being better able to reallocate attention when effectively encountering negative information (faster disengagement from stimuli), or both. Nevertheless, based on the significant follow-up analyses to test the positivity effect with the free-viewing task, our results add to some extent to the notion that the positivity effect is more likely to occur in unconstrained viewing conditions [2]. Moreover, it is interesting to notice that positive gaze patterns in older adults have been found in a prior study of our lab [12] using the engagement-disengagement task in participants with increased sad mood after a stress induction. However, when participants experienced decreases in calmness after the stress induction, the opposite pattern emerged, and participants moved attention slower away from sad faces. This suggests that older adults tend to show the positivity effect in situations where either no cognitive constraints are present or when cognitive constraints are present and emotion regulation is necessary, but not when cognitive constraints are present in absence of negative mood inductions or when too much stress is present. However, given that no comparison with a younger adult group was used in this study [12], this idea of older adults adaptively applying the attentional deployment strategy needs to be further investigated.

Furthermore, in literature [2] it has been suggested that the positivity effect might occur gradually over the lifespan, and this finding requires empirical evidence. It has been confirmed in a valence judgement study [10], but this was not yet investigated in an attention study. Our large age ranges in both age groups allowed us to examine within-group correlations between ageing and attentional indices. However, in both our studies, we failed to find significant correlations. This means that either the change occurs in a specific time in the lifespan, or perhaps more likely, that the gradual change in attention is rather very small (and smaller than the age-related changes in valence judgements) and can only be seen when using age groups with a very large age gap.

### Attention and personality in younger and older adults

In our exploration of the relationship between attention and personality, no compelling evidence was found in the older adults group for a trait-inconsistent pattern, nor for a trait-consistent pattern. Although most results were insignificant and often in opposite direction of what we expected, we did find in the first study that negative emotionality and conscientiousness were related to faster disengagement from sad faces. So, with negative emotionality some evidence was found for a trait-inconsistent pattern, but no such results were found with the other more negative trait, neuroticism. Moreover, no significant effect was found with negative emotionality in the second study. For conscientiousness, however, we did find similar results in the second study, with conscientiousness being related to shorter viewing time towards sad faces. It might be worthwhile in future studies to investigate the relationship between emotional information processing and more regulatory personality traits besides the affective traits.

Concerning our younger adult group, interestingly, we did not find, in either study, robust evidence for a trait-consistent attentional viewing pattern. This may be in part due to the effect being rather small in non-clinical samples [16], requiring very large sample sizes to find such significant effects. However, unlike in previous studies, the directions of the relevant correlations in study 1 were also mixed and not always in the expected directions.

One possible explanation for not replicating these patterns is that we recruited a more diverse sample from different educational levels, who were older (*M*age = 32 and 31) than the student samples in previous studies [15, 16, 17] (*M*age = between 19.40 and 23.21). It might be that the relationship between personality and attention already changes early in the lifespan. Longitudinal designs are needed to bring more clarity. Further, the role of education as a moderating factor in this relationship has to our knowledge not yet been investigated. A final difference with other studies is that we screened for psychopathology to ensure a non-clinical sample, whereas other studies’ samples did not perform such a screening [15, 17, 18]. A systematic review [39] indicates that university students experience substantially higher rates of depression compared to the general population. It might thus be that in student samples some of the participants with higher avoidance temperament or neuroticism, had (sub)clinical levels of depression (or other forms of psychopathology), which may make attentional bias for negative information more outspoken. An exception is the study of Paelecke and colleagues [16]. They removed participants scoring above the clinical cut-off on the Beck Depression Inventory. Interestingly, they did not find significant relationships between the traits neuroticism and avoidance temperament and attention to unpleasant stimuli either. Only when they depleted participants’ cognitive resources by making their task more challenging, significant relationships emerged. So it might be that in a strict non-clinical sample, persons scoring higher on these more negative personality traits can completely compensate for their tendency to look at negative information, unless they get into more challenging situations. As [40] suggested in their review; up to moderate levels, neuroticism might be related to improved conflict monitoring and more cognitive control. Since we did not use a very challenging task, this could explain why we did not find evidence for trait-congruent attentional patterns with negative traits in our non-clinical samples. However, it does not explain why we also did not replicate evidence for extraversion being related to giving more attention to positive information.

Finally, different kinds of processing might also play a role in the occurrence of biases. Attention can be divided into three categories: alerting (being ready to process information), orienting (selecting information or moving attention towards a stimulus), and executive attention (resolving conflict in attention or for example focusing on one stimulus while ignoring the irrelevant information [41]). Our two experimental tasks can be classified in the orienting attention category. On contrary, emotional Stroop tasks used in other studies [18, 16] can be placed in the executive category. Moreover, it has been argued that the emotional Stroop task cannot be seen as a valid attentional measure (pp. 21–22 in [42]), so previous results should be handled with caution. Other tasks used in previous studies [15, 18] were lexical decision tasks, word fragmentation, word recognition, and word recall, thus all tasks in which top-down processing (e.g. memory) likely plays a larger role. This suggests that personality traits play a larger role in the top-down processing (interpretation) of emotional information than they do in bottom-up processing (like attentional orienting).

### The positivity effect and the role of personality

Our results indicate that personality traits most likely do not have an effect on the occurrence of the positivity effect. In study 1 we found two significant relationships between personality traits and attention in our older adult group, but Fisher *r*-to-*z* transformations showed that these correlations did not differ significantly from the correlations in the younger adult group. In study 2, we did find a trend significant interaction effect with conscientiousness, indicating that this trait influences looking at sad faces in older adults, whereas this is not the case in younger adults. However, based on this one difference between younger and older adults, we cannot claim that personality traits generally influence the occurrence of the positivity effect.

### Limitations

The positivity effect is regarded as an age-valence interaction, where an older adult would give more attention to positive or less attention to negative stimuli than a younger adult, when these are presented simultaneously [2]. In both our experiments we actually never presented positive and negative stimuli simultaneously, but always calculated the time towards/away/spent on an emotional stimuli relative to a neutral stimulus that was presented at the same time. The reason we opted to present our emotional stimuli in combination with a neutral stimulus, is because we considered it more relevant for everyday life (i.e. more likely that someone encounters a situation in which there are either strong negative stimuli or strong positive stimuli together with more not-emotions-evoking stimuli). Nevertheless, we did succeed in finding evidence for an age-valence interaction even with this composition of stimuli in a free-viewing condition.

Another limitation is that our sample size may have been too restricted to gain enough power to find relationships with small effect-sizes between personality and attentional bias. Nevertheless, the directions of our relevant results were also quite mixed, so probably even in larger samples the expected directions would not have been confirmed. Also, given that previous studies with larger samples not always found significant results or results with very small effect sizes [15, 16, 17], one might question whether the relationship between personality and attentional biases in non-clinical samples would have many practical implications for individuals in daily life. Unfortunately, our small sample sizes also did not allow us to investigate the interaction between affective and regulatory traits in predicting information processing. This might be an interesting question for future research, given that there may be a more complex relationship between different personality traits and emotional information processing.

A final limitation of our study is that we did not have a condition in which more severe cognitive constraints were present [16]. It would be interesting to examine whether in general samples screened for psychopathology, individuals scoring higher on negative personality traits would only show the negativity bias under situations depleting their cognitive/emotional resources, but not under normal situations.

## General conclusion

We only found very limited evidence for a positivity effect in older adults that should be interpreted with caution. The effect could only be observed in a free-viewing condition and not in an experiment with attentional constraints. More specifically, we found some indications for a positivity effect in older adults as indexed by less attention to negative stimuli as compared to younger adults. Based on our cross-sectional design, it seems unlikely that personality has an effect on the occurrence of the positivity effect in attention. Further research is needed to determine other influencing factors and identify circumstances under which older adults apply this positive gazing strategy. Moreover, the relationship between neuroticism and extraversion and attentional processing of emotional information in both older and younger adults seems to be far smaller than expected based on the theoretical assumptions. Also, it might be worthwhile to also include other personality traits, for example more regulatory traits, given that we did find relationships between conscientiousness and less attention towards negative stimuli, albeit only in our older sample.

A strength of our study was that our findings are based on a more community-representative sample compared to students-only samples. However, although cross-sectional designs are the norm in research on the positivity effect, a longitudinal design would give more insight on how attention and the relation between attention and personality evolves over the lifespan.

## Supporting information

S1 Fig Visualization of the trend significant interaction effect in study 2. (DOCX)
