# Supplementary figures and images for: The relationship between attentional processing of emotional information and personality: A comparison between older and younger adults
Source: PLoS One. 2019 May 23;14(5):e0217382. doi: 10.1371/journal.pone.0217382 (PMC6532912; doi:10.1371/journal.pone.0217382)

Supplement: Interaction effect study 2


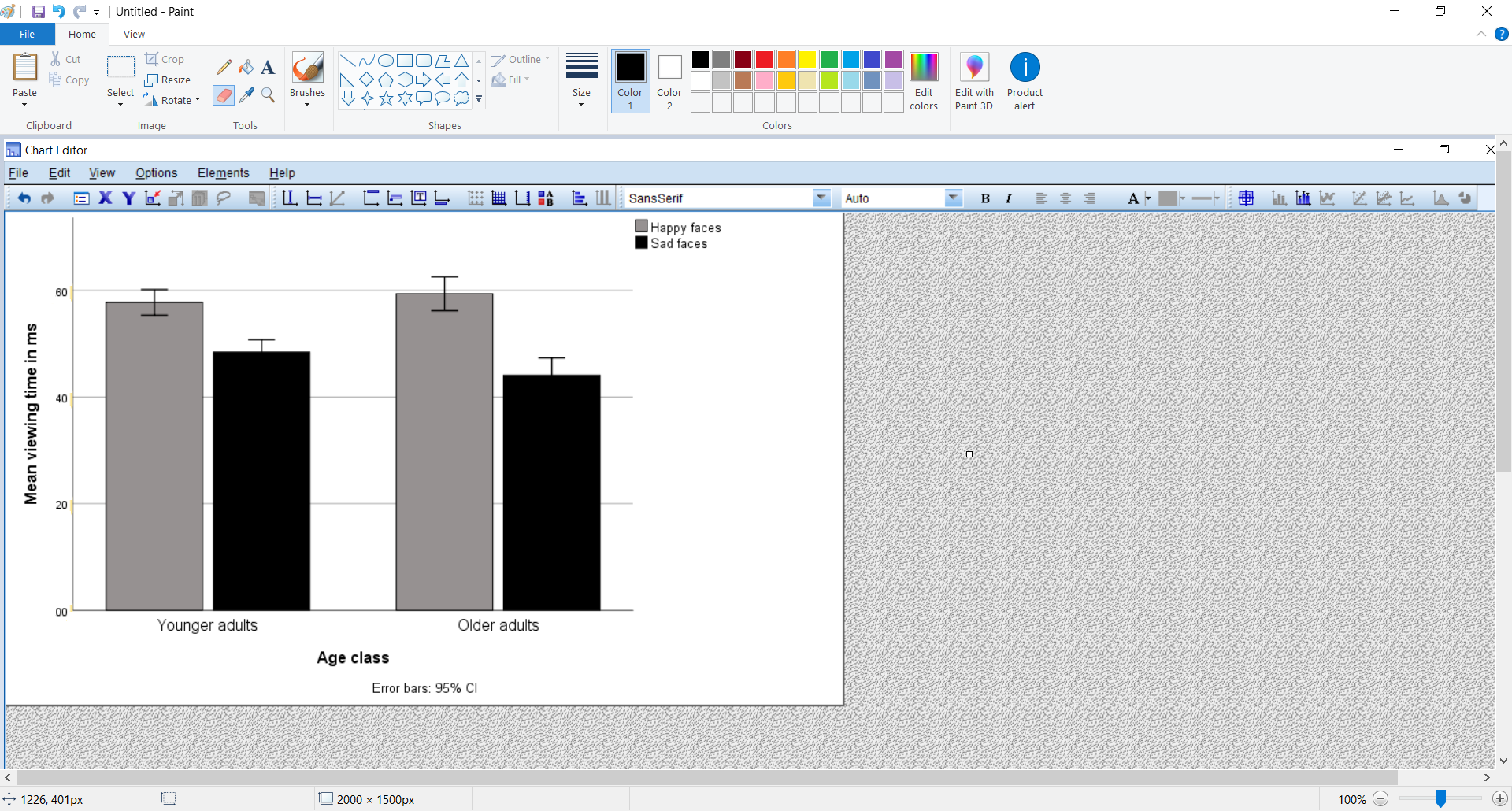

Supplement: S1 Fig — (DOCX) [file pone.0217382.s001.docx]
